# Energy-Based Multiresolution Analysis of FBG-Measured Strain Responses for Void Detection in Curved Pressure Vessel Structures Under Guided Wave Excitation

**DOI:** 10.3390/s26092768

**Published:** 2026-04-29

**Authors:** Ziping Wang, Napoleon Kuebutornye, Xilin Wang, Qingwei Xia, Alfredo Güemes, Antonio Fernández López

**Affiliations:** 1 Faculty of Civil Engineering and Mechanics, Jiangsu University and National Center for International Research on Structural Health Management of Critical Components, Zhenjiang 212013, China; napoleon.kuebutornye@bolgatu.edu.gh (N.K.); 2212323012@stmail.ujs.edu.cn (Q.X.); 2 Bolgatanga Technical University, Bolgatanga P.O. Box 767, Ghana; 3 Shenzhen International Graduate School, Institute of Ocean Engineering, Tsinghua University, Shenzhen 518055, China; wang.xilin@sz.tsinghua.edu.cn; 4 Yangzhou Ruitan Technology Co., Ltd., Yangzhou 225000, China; 5 Department of Aeronautics, Polytechnic University of Madrid, 28040 Madrid, Spain; alfredo.guemes@upm.es (A.G.); antonio.fernandez.lopez@upm.es (A.F.L.)

**Keywords:** structural health monitoring, guided wave excitation, fiber Bragg grating sensors, multiresolution wavelet analysis, energy-based diagnostics

## Abstract

Reliable detection of internal defects in pressure vessel structures remains essential for structural safety and condition-based maintenance. This study presents a low-complexity structural health monitoring framework based on fiber Bragg grating (FBG) sensing and multiresolution wavelet analysis for void detection in curved pressure vessel structures under guided wave excitation. Guided waves are introduced using piezoelectric actuators, while the FBG sensors capture the resulting strain-induced wavelength variations. Due to the limited bandwidth of the optical interrogator, the recorded signals represent the strain envelope response associated with guided wave interaction rather than the resolved ultrasonic carrier waveform. To characterize defect-induced changes, the acquired signals are analyzed using continuous wavelet transform (CWT) for time–frequency interpretation, and discrete wavelet transform (DWT) and wavelet packet transform (WPT) for energy-based multiresolution feature extraction. Experimental results show that void defects lead to consistent redistribution of wavelet-domain energy and increased non-stationarity in the measured strain responses. These trends are further supported by finite-element simulations, which reproduce similar energy redistribution patterns between intact and damaged cases. The proposed framework provides a physically interpretable and computationally efficient approach for defect detection using low-bandwidth FBG sensing, without reliance on high-speed acquisition or data-intensive learning models. The results demonstrate the feasibility of using energy-based multiresolution analysis of FBG strain signals for practical and scalable structural health monitoring of pressure vessel systems.

## 1. Introduction

**Figure 1:**
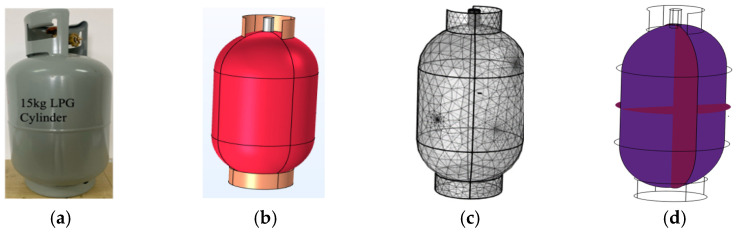
Experimental and numerical configuration of the pressure vessel structure: (**a**) 15 kg LPG pressure vessel used in the experiment, (**b**) geometry, (**c**) mesh, and (**d**) guided wave propagation model.

Early approaches to the structural health monitoring (SHM) of pressure vessels (PVs), as illustrated in Figure 1a, primarily relied on visual inspection conducted by engineers and skilled personnel during construction and early service stages. Although widely adopted, visual inspection is inherently subjective and limited to surface visible defects, making it unsuitable for early damage detection or continuous monitoring of safety critical pressure vessel structures (PVSs). The 1950s to 1960s marked a major transition with the development of non-destructive testing and evaluation (NDT/E) techniques aimed at detecting subsurface material and structural damage, including fatigue cracking in metallic components and delamination in composite structures [1]. These methods significantly improved inspection reliability; however, they remained periodic, labor-intensive, and dependent on operator expertise.

In the 1980s, acoustic emission (AE) techniques emerged as a promising approach for PV monitoring. Ohtsuka et al. [2] demonstrated the feasibility of using AE signals for defect detection in pressure vessels, enabling continuous monitoring of damage evolution. To enhance robustness under noisy operating conditions, adaptive signal processing techniques such as adaptive singular value decomposition (ASVD) were later introduced [3,4]. Subsequent studies further advanced AE-based monitoring, including investigations of deformation mechanisms in composite pressure vessels [5] and crack growth monitoring in metallic structures [6,7,8], establishing AE as a viable technique for in situ structural assessment. During the 1990s, research emphasis shifted toward guided ultrasonic wave-based SHM, accompanied by advances in signal processing and early networked monitoring systems. Guided waves gained prominence due to their high sensitivity to structural discontinuities and their ability to inspect large areas using a limited number of sensors [9,10]. These characteristics significantly improved detection capability and supported the development of real-time structural monitoring approaches.

In the 2000s and beyond, substantial technological progress further expanded SHM capabilities. Research extended toward Internet of Things (IoT) enabled monitoring systems, facilitating real-time data acquisition, transmission, and analysis [11,12]. Parallel developments focused on diagnostic measurement, information condensation, and damage identification across a wide range of engineering structures, including aircraft, wind energy systems, bridges, offshore platforms, and mechanical components [13,14]. In addition, guided ultrasonic waves were increasingly applied to composite and hybrid structures for detecting defects such as disbonding and delamination through advanced modeling and signal processing techniques [15]. Guided wave-based SHM methods are commonly categorized into passive and active approaches. Passive methods, such as AE, rely on damage-induced acoustic activity but often suffer from limited repeatability and controllability. In contrast, active methods employ controlled excitation, enabling repeatable measurements, improved signal interpretation, and enhanced defect localization. As a result, active guided wave techniques are widely regarded as effective solutions for large-area monitoring of pressure vessel structures [16].

FBG sensors have attracted growing interest in structural health monitoring because they are lightweight, immune to electromagnetic interference, suitable for harsh environments, and capable of high-sensitivity strain measurement. In guided wave applications, FBGs provide an attractive optical alternative to conventional electrical sensors, although their effectiveness depends strongly on interrogation bandwidth, sensor orientation, bonding quality, and the relationship between the measured optical response and the underlying structural wavefield. This makes them particularly relevant for low-complexity monitoring scenarios where sensing transparency and environmental robustness are important [17,18,19]. Recent studies have further highlighted the versatility of FBG-based sensing in structural monitoring, including damage assessment of steel structures under coupled loading conditions and integration with other optical sensing strategies, which reinforces the suitability of FBG technology for practical SHM environments [20].

Despite the progress of guided wave structural health monitoring, several challenges remain for practical pressure vessel applications. Many existing approaches rely on dense sensor layouts, high-speed acquisition systems, or carrier-resolved waveform analysis, which increase hardware complexity and reduce practicality for low-cost deployment [21,22,23]. In addition, most reported FBG-guided wave studies focus on flat or relatively simple geometries, while curved pressure vessel structures and internal void-type defects remain less explored. Existing studies also tend to emphasize qualitative time–frequency visualization, with fewer efforts devoted to physically interpretable multiresolution energy diagnostics under bandwidth-limited sensing conditions [24,25,26,27,28,29,30].

Unlike conventional guided wave monitoring systems that depend on the direct acquisition of the ultrasonic carrier waveform, the present study investigates whether defect-sensitive information can still be extracted from low-bandwidth FBG strain response signals recorded by a 2 kHz interrogator under 100 kHz guided wave excitation. The main contribution is therefore not only the use of FBG sensing itself, but the demonstration that multiresolution wavelet-energy features can provide physically interpretable and computationally efficient indicators of void presence in a curved pressure vessel structure, even when the ultrasonic carrier is not directly resolved. To support this interpretation, the study combines controlled experiments with qualitative finite-element analysis and statistical validation of selected wavelet-energy features [31,32].

Therefore, this study makes three main contributions. First, it establishes a sensing paradigm in which low-bandwidth FBG interrogators are used to capture strain envelope responses induced by guided wave excitation, eliminating the need for high-speed ultrasonic acquisition systems. Second, it introduces a unified multiresolution energy-based diagnostic framework that integrates continuous wavelet transform (CWT)-based time–frequency interpretation with discrete wavelet transform (DWT) and wavelet packet transform (WPT) energy features, enabling the physically interpretable characterization of defect-induced signal changes. Third, the proposed framework is validated on a curved pressure vessel geometry, demonstrating that meaningful defect-sensitive features can be extracted from single-channel FBG measurements while reducing sensing complexity.

## 2. Experimental Methodology and Specimen Description

**Figure 2:**
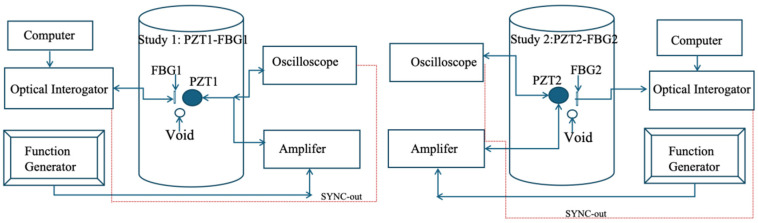
Schematic diagram of the experimental configuration showing the two alternative actuator–sensor paths used in the study, Study 1 (PZT1-FBG1) and Study 2 (PZT2-FBG2). Both configurations were tested separately under the no-void and void conditions using the same excitation, interrogation, and synchronization arrangement.

Experiments were conducted on a 15 kg liquefied petroleum gas (LPG) steel cylinder [33], selected as a representative curved, thin-walled pressure vessel structure (PVS) used in practical applications. Two structural conditions were investigated: (i) a no-void condition representing the intact state, and (ii) a void condition in which an artificial round void with a diameter of 4 mm and a depth of 1.8 mm was introduced to create a controlled internal discontinuity. This design enabled direct comparison of strain responses under identical excitation and acquisition conditions. Two FBG sensors (FBG1 and FBG2) were surface-mounted on the external wall of the cylinder using high-strength epoxy to ensure effective strain transfer. Guided wave excitation was generated using two PZT-5H piezoelectric transducers. Two measurement configurations were considered to examine sensitivity under different wave paths. In Study 1, PZT1 acted as the actuator and FBG1 as the receiver; in Study 2, PZT2 acted as the actuator and FBG2 as the receiver, as illustrated in Figure 2. Each configuration represents a single-sensor measurement scenario under a distinct actuator–sensor path, and both configurations were tested under the no-void and void conditions.

The PZT actuators were driven using a high-voltage amplifier (ATA-2021H, Xi’an Aigtek Electronic Technology Co., Ltd., Xi’an, China). Excitation signals and trigger timing were monitored using a Tektronix 4-Series mixed-signal oscilloscope (Tektronix, Inc., Beaverton, OR, USA). The FBG wavelength responses were recorded using a Micron Optics SM130 optical interrogator (Micron Optics, Inc., Atlanta, GA, USA) operating at a sampling rate of 2 kHz.

### 2.1. Excitation and Acquisition Settings

**Figure 3:**
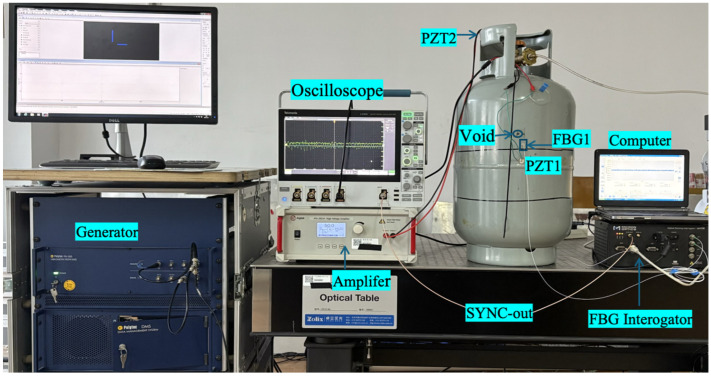
Experimental setup for guided wave excitation and FBG signal acquisition.

Guided wave excitation was applied using a five-cycle tone-burst signal with a central frequency of 100 kHz, while the FBG wavelength response was recorded using an optical interrogator operating at 2 kHz. Because the interrogator sampling rate is far below the ultrasonic carrier frequency, the acquired signal does not represent a carrier-resolved guided wave waveform in the conventional ultrasonic sense as shown in Figure 3. Instead, the recorded output should be interpreted as a low-bandwidth strain response signal that reflects the effective temporal organization of local structural strain induced by guided wave transmission, structural curvature, and defect interaction.

In other words, the interrogator captures envelope-level and slow-varying response behavior within its observable bandwidth rather than phase-resolved 100 kHz oscillations. Accordingly, the present framework does not rely on ultrasonic phase, direct time-of-flight estimation, or cycle-level spectral interpretation. Instead, it analyzes how defect presence modifies the measured strain response organization through time–frequency localization and wavelet-domain energy redistribution. This distinction is fundamental to the study, because the objective is not to reconstruct the ultrasonic waveform, but to determine whether meaningful defect-sensitive information remains embedded in the low-bandwidth FBG response and can be extracted through interpretable multiresolution features.

### 2.2. Numerical Simulation

A three-dimensional transient finite-element model was developed in COMSOL Multiphysics 6.2 to support the physical interpretation of the experimentally observed response differences between the intact and voided conditions. The geometry was based on the 15 kg LPG steel cylinder used in the experiments, as shown in Figure 1b. The defect was represented as a round void with a diameter of 4 mm and a depth of 1.8 mm, as shown in Figure 1c. The coupled electromechanical model included the elastic wave, time explicit, solid mechanics, and electrostatics interfaces, together with piezoelectricity multiphysics coupling to represent guided wave actuation by the PZT transducer.

The computational domain was discretized using a free tetrahedral mesh. A coarse-to-moderate mesh was used in the general vessel domain, while locally refined mesh regions were introduced around the PZT and FBG sensing locations, the void region, and other areas expected to experience stronger wave interaction in order to better capture local response variations. The global mesh setting employed a predefined normal size distribution with maximum and minimum element sizes of 58.5 mm and 10.5 mm, respectively. In addition, the local mesh control calibrated for time-explicit wave propagation was applied in selected domains. The transient analysis was performed over 0 to 1 ms, with results sampled at 1 µs intervals.

**Figure 4:**
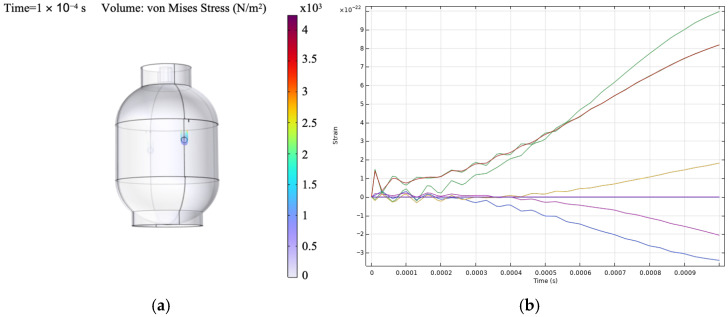
Numerical outputs used for physical interpretation, (**a**) electric potential distribution on the actuator region under piezoelectric excitation, (**b**) extracted strain response histories at selected virtual probe locations used as transient response observables for qualitative comparison of intact and defective conditions.

Free boundary conditions were assigned in the structural and elastic wave interfaces, while the electrostatic domain used terminal and ground assignments for piezoelectric excitation. Two virtual sensing locations were defined using point probes corresponding to the experimental sensor positions. At both probe locations, the extracted response quantity was the average spatial value of the ZZ-component of the strain tensor (solid.eZZ), evaluated over the selected probe regions. A boundary coordinate system was defined with tangential and normal directions referenced to the global Cartesian frame, with the tangential construction based on the *z*-axis, so that the extracted strain component remained consistent with the chosen sensor-aligned direction. Guided wave excitation was modeled using a surface-mounted PZT-5H actuator represented through fully coupled piezoelectric constitutive relations. A five-cycle tone-burst signal at 100 kHz was applied as a voltage boundary condition to match the experimental excitation, as indicated in Figure 1d. The numerical model was used primarily to reproduce qualitative differences in strain response organization between the intact and defective states rather than exact carrier-resolved waveform agreement. The extracted strain histories shown in Figure 4b are not interpreted as carrier-resolved ultrasonic waveforms. Instead, they are treated as transient response observables used to compare the relative organization of the intact and defective cases within the adopted numerical framework. This distinction is important because the present study focuses on qualitative agreement in defect-related energy redistribution rather than exact waveform reproduction. Similar use of transient wavefield observables and explicit finite-element setup descriptions has been reported in prior guided wave numerical studies [34].

Two types of outputs were used to support interpretation. First, the spatial distribution of electric potential on the actuator surface, shown in Figure 4a, provides qualitative validation of the applied excitation and electromechanical coupling. Second, the extracted strain time histories at the virtual probe locations, shown in Figure 4b, exhibit spatial variability in response amplitude and temporal behavior. Some locations show stronger dynamic responses due to wave propagation and defect interaction, while others exhibit weaker responses, indicating reduced coupling with the wavefield. These variations support the use of localized strain measurements as defect-sensitive observables and are consistent with the experimentally observed energy redistribution patterns.

### 2.3. Mathematical Theory and Principles of the Study

The sensing principle adopted in this study differs from that of conventional high-speed guided wave acquisition. In a carrier-resolved system, the measured signal preserves the ultrasonic waveform directly and supports phase-sensitive analysis, arrival-time estimation, and mode-dependent interpretation. In the present setup, however, the interrogator bandwidth is much lower than the excitation frequency. As a result, the measured FBG output is treated as a bandwidth-limited strain response observable rather than a direct ultrasonic waveform. The analysis therefore focuses on how defect presence modifies the temporal organization and multiscale energy distribution of this observable.

Guided wave excitation induces a transient strain field on the surface of the pressure vessel. This strain causes a shift in the Bragg wavelength of the FBG sensor. Under isothermal conditions, the relationship between axial strain and wavelength shift is expressed as

(1) $$\Delta \lambda_{B}/\lambda_{B}=(1-P_{e})\varepsilon$$

where $\Delta \lambda_{B}$ is the Bragg wavelength shift, $\lambda_{B}$ is the initial Bragg wavelength, *P_e_* is the effective photoelastic coefficient, and *ε* is the axial strain.

### 2.4. Continuous Wavelet Transform (CWT)

Guided wave-induced strain responses are inherently non-stationary due to dispersion, mode interaction, and defect-induced scattering. To capture these characteristics, the continuous wavelet transform (CWT) is employed. For a signal *x*(*t*), the CWT is computed as [35]

(2) $$W(a,B)=\int_{-\infty }^{\infty }x(t)\psi^{*}\left(\frac{t-b}{a}\right)dt$$

where *a* is the scale parameter related to frequency, *b* is the time translation parameter, u(⋅) is the mother wavelet, and ${\left(\cdot \right)}^{*}$ denotes complex conjugation. The magnitude of the wavelet coefficients provides a time–frequency representation scalogram, which enables the visualization of energy distribution, temporal localization, and non-stationary behavior associated with defect presence.

### 2.5. Discrete Wavelet Transform (DWT)

To obtain compact and quantitative features, the discrete wavelet transform (DWT) is used to decompose the signal into multiple resolution levels corresponding to different frequency bands [36]:

(3) $$x(t)=\sum_{k}A_{J,k\phi J,k}(t)+\sum_{j=1}^{J}\sum_{k}D_{j,k\psi j,k}(t)$$

where *A_J,K_* denotes the approximation coefficients at level *J*, *D_j,k_* denotes the detail coefficients at level j,ϕ,ψ denote the scaling and wavelet functions, respectively.

(4) $$E_{j}=\sum_{k}{\left|D_{j,k}\right|}^{2}$$

To allow comparison across signals, the energy is normalized:

(5) $${\tilde{E}}_{j}=\frac{E_{j}}{\sum_{j=1}^{J}E_{j}}$$

Changes in normalized energy distribution reflect defect-induced modifications in the strain response.

### 2.6. Wavelet Packet Transform (WPT) and Node Energy

The WPT provides finer frequency resolution by decomposing both approximation and detail components. For a wavelet packet node, the energy is defined as

(6) $$E_{n}={\sum_{k}\left|C_{n,k}\right|}^{2}$$

where *C_n,k_* are the wavelet packet coefficients. The normalized energy is given by

(7) $${\tilde{E}}_{n}=\frac{E_{n}}{\sum_{n=1}^{N}E_{n}}$$

where *N* is the number of terminal nodes. This representation is useful for detecting fine-band energy shifts that can occur when guided waves interact with localized voids [37].

### 2.7. Energy-Based Damage Interpretation

Void defects influence guided wave propagation through scattering, attenuation, and mode interaction. In the proposed framework, these effects manifest as the redistribution of signal energy across wavelet scales and frequency bands. CWT provides qualitative insight into time–frequency behavior, while DWT and WPT offer physically interpretable energy-based features. Together, these methods enable a multiresolution diagnostic framework suitable for analyzing FBG-measured strain envelope responses in pressure vessel structures.

## 3. Results and Discussion

### 3.1. Preprocessed FBG Strain Responses

**Figure 5:**
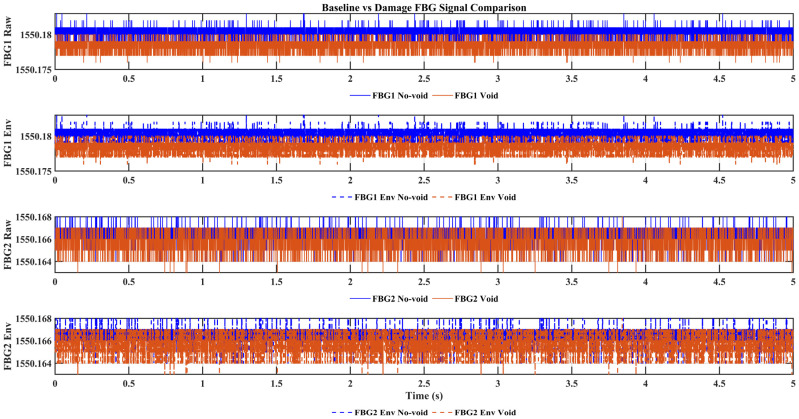
Comparison of preprocessed FBG strain responses under no-void and void conditions at the two sensing locations, (FBG 1 Raw) FBG1 preprocessed signal, (FBG Env) FBG1 envelope response, (FBG 2 Raw) FBG2 preprocessed signal, and (FBG 2 Env) FBG2 envelope response.

Figure 5 compares the preprocessed FBG responses under the no-void and void conditions for the two actuator–sensor paths. Although the interrogator does not resolve the ultrasonic carrier waveform, the recorded strain response traces still exhibit condition-dependent differences in temporal organization. Relative to the intact state, the void condition shows more irregular envelope evolution and stronger local variations over time, indicating that the defect modifies the effective transmitted response measured at the sensing location. These observations motivate the subsequent multiresolution analysis, which provides a more structured assessment of how these differences are redistributed across scales and frequency bands within the observable bandwidth.

These visual differences are also supported by simple window-based signal descriptors. As summarized in Table 1, the void condition is associated with greater signal irregularity, particularly in the envelope-based variability measures. The most consistent increase is observed in the envelope standard deviation, which rises from 0.5842 to 0.6262 for FBG1 and from 0.6587 to 0.7691 for FBG2, supporting the interpretation that void presence produces stronger local fluctuations and less stable response organization.

It should be emphasized that the traces shown in Figure 5 are not carrier-resolved ultrasonic waveforms. Because the optical interrogator operates at 2 kHz while the guided wave excitation is centered at 100 kHz, the recorded signals represent bandwidth-limited strain response observables rather than direct cycle-level representations of the excitation signal. Accordingly, the apparent difference between the excitation form and the measured traces does not indicate inconsistency in the sensing process, but reflects the low-bandwidth response nature of the adopted FBG interrogation system. The displayed amplitude resolution is also constrained by the optical interrogator output and the plotting scale used for visualization. For this reason, the interpretation does not rely on fine quantized amplitude detail in the raw traces, but on the more robust multiresolution organization of the measured response in the time–frequency and wavelet-energy domains.

### 3.2. Time–Frequency Characteristics from CWT Scalograms

**Figure 6:**
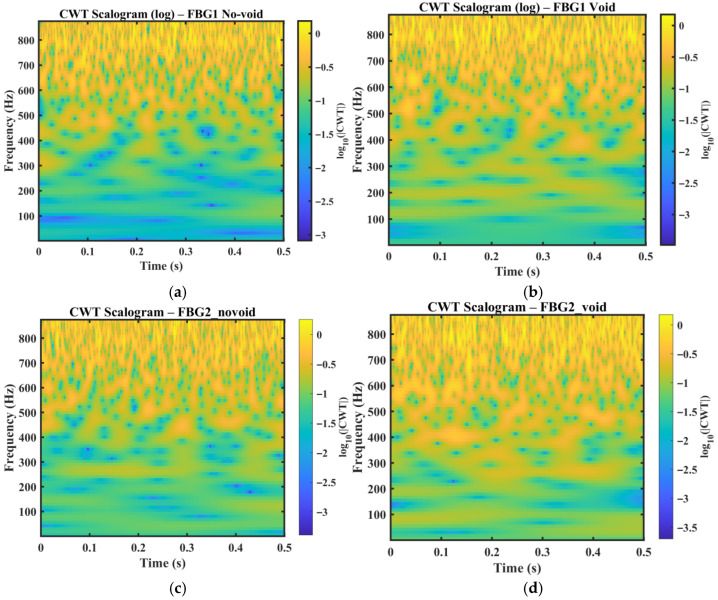
CWT scalograms of the FBG wavelength responses for the no-void and void cases at the two sensing locations, (**a**) FBG1 no-void, (**b**) FBG1 void, (**c**) FBG2 no-void, and (**d**) FBG2 void.

Figure 6 present the logarithmic continuous wavelet transform (CWT) scalograms of the measured FBG wavelength responses for the no-void and void conditions at the two sensing locations, FBG1 and FBG2. The resulting time–frequency maps show broadband signal components within the bandwidth of the optical interrogator. Under the no-void condition, the energy distribution remains relatively smooth and temporally stable, indicating a more repeatable strain envelope response under nominal wave propagation conditions. In contrast, the void condition exhibits increased non-stationarity, characterized by localized energy concentrations and intermittent patches distributed across multiple frequency bands. These features are consistent with defect-induced modifications in the measured response, where interaction between the guided wavefield and the void introduces additional scattering, attenuation, and mode interaction, leading to the time-varying redistribution of signal energy. The present study does not invoke nonlinear ultrasonic mechanisms to explain these localized energy concentrations. Instead, the observed CWT patterns are interpreted within a linear guided wave framework in which defect-induced scattering, attenuation, dispersion, and mode interaction reorganize the measured low-bandwidth strain response.

To provide quantitative support for these visual observations, simple descriptors were also derived from the CWT scalograms, including scalogram entropy, energy concentration, and the high- or low-band energy ratio in Table 2. The void condition exhibits a higher high- or low-band energy ratio than the corresponding no-void condition for both sensing paths, increasing from 0.022325 to 0.026720 for FBG1 and from 0.025690 to 0.026485 for FBG2. This supports the interpretation that the defect redistributes more energy toward higher observable frequency bands within the interrogator-accessible response. A slight increase in scalogram entropy is also observed for the FBG1 void case relative to the no-void case, indicating a modest increase in time–frequency irregularity under defect interaction.

A comparison of the two sensing locations further shows that the magnitude and visibility of these perturbations differ between FBG1 and FBG2. This difference reflects the combined effects of wave path, structural curvature, and local strain coupling at each sensing position. In the present dataset, the void case measured at FBG2 exhibits more pronounced localized energy perturbations than the corresponding no-void case. This suggests that defect sensitivity is not governed solely by sensor proximity to the defect, but also by how scattered wave energy is redistributed along the curved vessel wall and coupled into the surface strain measured by the FBG sensor.

Overall, the CWT scalograms provide an interpretable view of damage-related non-stationary behavior that is not readily visible in the raw wavelength traces. Although the CWT results are used here primarily for qualitative interpretation, they provide clear evidence that the void condition alters the time–frequency structure of the measured strain response and thereby motivates the subsequent use of DWT- and WPT-based energy features for quantitative discrimination.

### 3.3. DWT-Normalized Energy Distribution

**Figure 7:**
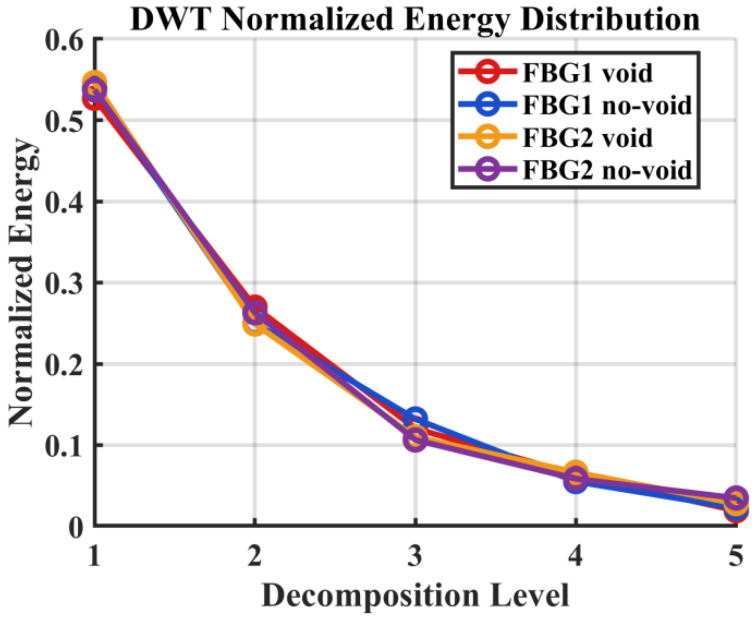
Normalized DWT energy distribution across five decomposition levels for FBG1 and FBG2 under void and no-void conditions.

For interpretive clarity, the five-level DWT decomposition can be related approximately to the observable frequency bands of the recorded signal based on the interrogator sampling rate of 2 kHz. With a Nyquist frequency of 1 kHz, the detail levels correspond approximately to the following bands, namely D1 = 500–1000 Hz, D2 = 250–500 Hz, D3 = 125–250 Hz, D4 = 62.5–125 Hz, and D5 = 31.25–62.5 Hz, while the approximation component A5 represents frequencies below 31.25 Hz, as shown in Table 3. In this study, the decomposition depth was set to J = 5, which provided sufficient multiscale resolution while maintaining stable coefficient estimates for the available signal length and the bandwidth-limited nature of the recorded FBG response. Since the interrogator does not resolve the 100 kHz carrier waveform directly, these decomposition levels should be interpreted as relative frequency partitions of the low-bandwidth FBG strain response signal rather than as direct representations of the ultrasonic excitation frequency. The DWT results represented in Figure 7 shows that most of the observable signal energy remains concentrated in the lower decomposition bands, particularly D4–D5 and A5, which is consistent with the bandwidth-limited nature of the recorded response. Relative to the intact condition, the void case produces a modest but repeatable redistribution of normalized energy toward higher decomposition levels. Within the present framework, this should not be interpreted as a direct observation of the 100 kHz carrier, but rather as evidence that the defect increases the multiscale irregularity of the measured strain response signal within the interrogator-accessible bandwidth. This effect is more visible for the second actuator–sensor path, suggesting that defect sensitivity depends on the combined influence of wave path, vessel curvature, and local strain coupling.

### 3.4. WPT-Normalized Energy Distribution

**Figure 8:**
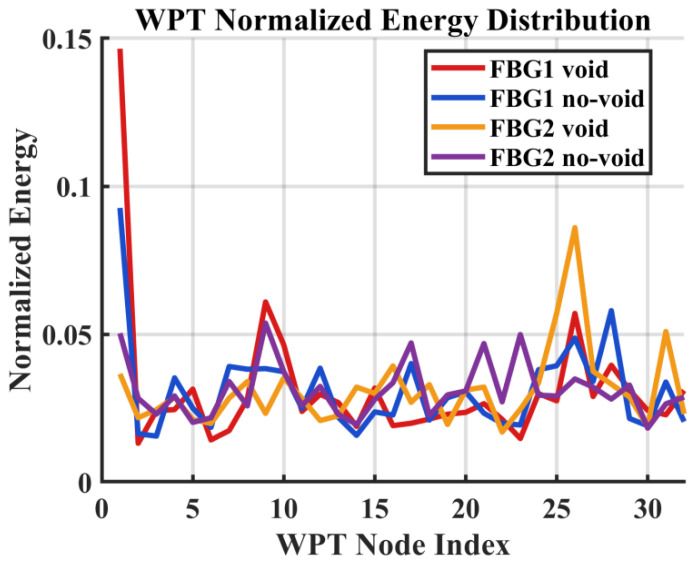
Normalized WPT node-energy distribution for FBG1 and FBG2 under void and no-void conditions.

The WPT analysis as shown in Figure 8 provides finer band partitioning of the same low-bandwidth strain response signal and shows that the void condition produces localized node-wise energy shifts relative to the no-void case. These changes are consistent with defect-induced reorganization of the measured response and complement the coarser trends observed in the DWT analysis. This interpretation is further supported by the statistical validation results in Table 1, where selected node- and level-based features exhibit significant separation between the no-void and void conditions. In particular, the WPT features, FBG2 WPT-N1 and FBG2 WPT-N3, show clear increases in mean normalized energy under the void condition, with *p*-values of 2.14 × 10^−6^ and 7.06 × 10^−6^, respectively, confirming that the observed node-wise energy changes are reproducible and statistically significant. The later statistical results therefore do not merely support the WPT interpretation in a general sense; they show that selected local node-energy increases are repeatable and significant across the adopted window-based observations.

### 3.5. Numerical Simulation Results

The numerical model was used to support the physical interpretation of the experimentally observed energy redistribution, rather than to reproduce absolute amplitudes or exact frequency values. Strain time histories were extracted at virtual probe locations corresponding to the experimental FBG positions, and the same multiresolution analysis framework was applied to the simulated responses, including CWT for time–frequency visualization and DWT/WPT for energy-based characterization.

**Figure 9:**
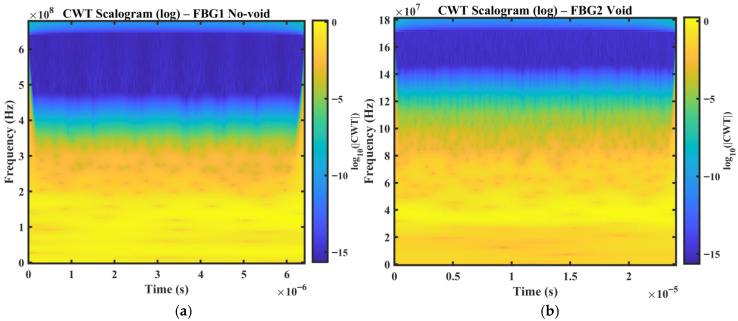
Numerical CWT scalograms (log magnitude) obtained from simulated strain responses at virtual FBG locations: (**a**) FBG1 no-void (intact condition), (**b**) FBG2 void (defect condition).

Figure 9a,b show representative CWT scalograms obtained from the simulated strain responses for an intact case (FBG1 no-void) and a defective case (FBG2 void), respectively. In the intact condition, the scalogram exhibits a smoother and more stable time–frequency energy distribution, consistent with guided wave propagation in the absence of a structural discontinuity. In contrast, the defective case shows attenuation and redistribution of energy, reflecting the influence of defect-induced scattering and mode interaction on the simulated strain response. The absolute frequency axes of the numerical scalograms should not be interpreted as directly equivalent to those of the experimental results, since they depend on discretization settings, numerical solver parameters, and modeling assumptions. The main point of comparison is therefore not absolute frequency alignment, but the relative change in the energy distribution between intact and voided conditions. In this respect, the simulation and experiment show the same qualitative trend. The presence of a void modifies the temporal and spectral organization of the strain response and produces measurable redistribution of signal energy.

The defect-related changes observed in the simulated responses are consistent with established guided wave interaction mechanisms, in which local discontinuities such as voids or notches modify wave transmission through scattering, attenuation, and mode interaction. In the present study, these effects are reflected in the redistribution of strain response energy across time–frequency bands rather than in exact carrier-resolved waveform features [38].

Hence, the numerical results seek to reinforce the experimental interpretation by showing that the observed wavelet-energy changes are consistent with the expected physical effects of defect interaction. The agreement between simulation and experiment therefore supports the use of multiresolution energy features as physically meaningful indicators of structural condition in the proposed sensing framework.

### 3.6. Simulated DWT and WPT Energy Features

**Figure 10:**
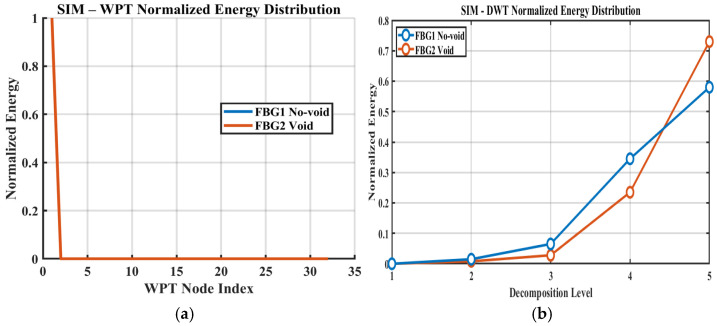
Numerical wavelet-energy features extracted from simulated strain responses: (**a**) WPT-normalized energy distribution across terminal nodes; (**b**) DWT-normalized energy distribution across decomposition levels (no-void vs. void).

To further quantify these observations, normalized DWT and WPT energy features were computed from the simulated strain responses. Figure 10a presents the WPT-normalized node-energy distribution, while Figure 10b shows the normalized DWT energy across the decomposition levels. Consistent with the experimental results, the void condition exhibits a clearer redistribution of energy across wavelet bands than the intact condition. In particular, the damaged case shows a shift in energy concentration across selected nodes and levels, indicating that the presence of a void modifies the structure of the strain response in a manner that is detectable through compact multiresolution features. These results support the interpretation that defect-induced changes are not limited to simple amplitude reduction, but involve broader reorganization of the signal energy across scales and frequency bands.

Overall, the simulated DWT and WPT results seem to reinforce the experimental findings and provide additional support for the physical meaning of the extracted energy-based indicators. The agreement between numerical and experimental trends suggests that void damage is more appropriately characterized as a redistribution and attenuation process in the measured strain response, rather than as a purely time-domain amplitude effect.

### 3.7. Statistical Validation of Selected Wavelet-Energy Features

To enable statistical comparison between structural states, each preprocessed signal was segmented into non-overlapping windows of 0.05 s, and the selected wavelet-energy features were extracted from each window. This procedure produced 100 window-based observations per condition for the reported comparisons. The mean values, standard deviations, and independent two-sample *t*-test results were then computed from these window-level feature values.

**Figure 11:**
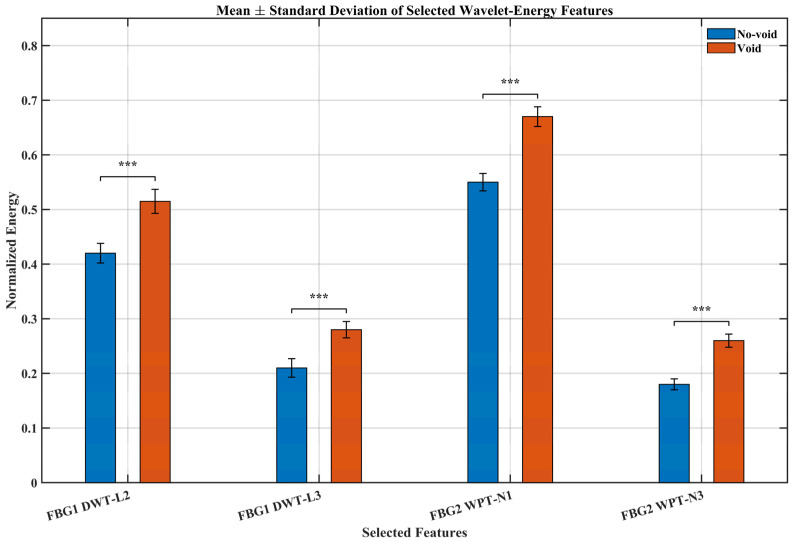
Mean ± standard deviation of selected wavelet-energy features for the no-void and void conditions. Error bars represent standard deviation. Statistical significance between paired no-void and void feature means is indicated by brackets, where *** denotes *p* < 0.001.

Figure 11 and Table 4 summarize the statistical behavior of the selected DWT and WPT energy features under the no-void and void conditions. For all four features, the void condition exhibits a clear increase in mean normalized energy relative to the no-void state. This trend is consistent with the earlier multiresolution results, which showed that defect presence leads to the redistribution of signal energy across wavelet scales and packet nodes. The standard deviations remain relatively small in both structural states, indicating limited within-condition variability and good repeatability of the extracted features across the adopted window-based observations. This suggests that the observed differences are not attributable to random fluctuations alone, but reflect stable changes in the measured strain response associated with the presence of the void.

The independent two-sample *t*-test results further confirm the statistical significance of these differences. All selected features yielded *p*-values below 0.001, demonstrating strong separation between the no-void and void conditions. Among the selected indicators, the WPT-based features show the largest mean shifts, indicating that fine-band energy redistribution is particularly sensitive to the presence of the defect. The DWT-based features also exhibit significant differences, confirming that both coarse and fine multiresolution energy measures provide useful discriminatory information.

Overall, the results in Figure 11 and Table 4 provide quantitative support for the discriminative capability of the proposed multiresolution wavelet-energy features. When considered together with the CWT, DWT, and WPT analyses, this statistical validation strengthens the conclusion that void damage produces consistent and measurable changes in the recorded FBG strain response.

### 3.8. Discussion and Implications for Pressure Vessel SHM

The combined experimental and numerical results indicate that single-FBG sensing under guided wave excitation, when interpreted as a low-bandwidth strain response measurement and analyzed through multiresolution wavelet methods, can distinguish between void and no-void conditions in the investigated curved pressure vessel structure. The CWT scalograms provide qualitative insight into damage-related changes in temporal response organization, while the normalized DWT and WPT energies offer compact quantitative indicators of structural condition. Together, these results show that useful defect-sensitive information can remain embedded in the interrogator-accessible response even when the ultrasonic carrier waveform is not directly resolved.

From a practical SHM perspective, the proposed framework offers several advantages. First, it reduces sensing complexity relative to dense multi-sensor layouts by demonstrating that a single-channel FBG measurement can still provide useful diagnostic information under the tested configurations. Second, the extracted wavelet-energy features are physically interpretable and computationally lightweight, making them suitable for low-complexity monitoring scenarios where transparency and efficiency are important. Third, the framework does not rely on large labeled datasets or data-intensive learning models, which improves its suitability for applications where limited experimental data are available.

At the same time, the present results should be interpreted within the scope of the investigated configurations. The study demonstrates feature sensitivity to the presence of a void under specific actuator–sensor paths, rather than full defect coverage across all possible locations and geometries. Detection performance may vary with defect size, position, structural complexity, and sensing arrangement. Nevertheless, the observed consistency between experimental and numerical trends suggests that multiresolution energy redistribution provides a useful physical basis for defect discrimination in curved pressure vessel structures.

Although the present study compares two actuator–sensor paths and demonstrates path-dependent sensitivity, it does not constitute a full sensor placement optimization analysis. Likewise, a formal signal-to-noise ratio evaluation was not performed in the current work. These aspects remain important directions for future study as the framework is extended toward deployment-oriented monitoring.

Overall, the findings support the feasibility of low-bandwidth FBG-based strain response monitoring for practical pressure vessel SHM. The study therefore provides a foundation for future work on extended sensor layouts, multiple defect scenarios, statistical validation under broader operating conditions, and integration with higher-bandwidth or hybrid sensing systems for enhanced diagnostic coverage.

## 4. Conclusions

This study presented a multiresolution wavelet-energy framework for void detection in a curved pressure vessel structure using single-FBG sensing under guided wave excitation. The key distinction of the proposed approach is that the FBG interrogator does not resolve the ultrasonic carrier waveform directly; instead, it records a low-bandwidth strain response signal whose temporal organization is influenced by guided wave transmission and defect interaction, from which defect-sensitive multiresolution energy features can still be extracted. By analyzing this response using CWT, DWT, and WPT, the study showed that void presence produces measurable changes in non-stationarity and wavelet-domain energy distribution. The extracted normalized energy features provided compact and physically interpretable indicators that consistently differentiated the void and no-void conditions under the investigated sensing paths.

Finite-element simulations reproduced the same qualitative energy redistribution trends observed experimentally, supporting the interpretation that the measured changes arise from defect-related scattering, attenuation, and mode interaction. This agreement between experiment and simulation strengthens the physical basis of the proposed sensing and analysis framework. Overall, the results demonstrate that low-bandwidth FBG measurements, when combined with multiresolution wavelet-energy analysis, provide a practical and low-complexity means of distinguishing void and no-void conditions in curved pressure vessel structures. Although the present study is limited to specific defect and sensing configurations, it establishes a useful foundation for future work on broader defect scenarios, expanded sensor layouts, higher-bandwidth interrogation, and hybrid sensing strategies.

## Figures and Tables

**Table 1:** Window-based signal-level descriptors of the preprocessed FBG responses under the no-void and void conditions. Statistics were computed from 100 non-overlapping windows of 0.05 s each.

Condition	*n*	RMS (Mean ± SD)	Variance (Mean ± SD)	Envelope Mean (Mean ± SD)	Envelope SD (Mean ± SD)
FBG1 No-void	100	0.9979 ± 0.0636	0.9930 ± 0.1275	1.2780 ± 0.0811	0.5842 ± 0.0567
FBG1 Void	100	0.9976 ± 0.0685	0.9891 ± 0.1338	1.2557 ± 0.0928	0.6262 ± 0.0455
FBG2 No-void	100	0.9962 ± 0.0864	0.9895 ± 0.1734	1.2351 ± 0.0932	0.6587 ± 0.1029
FBG2 Void	100	0.9957 ± 0.0930	0.9909 ± 0.1828	1.1707 ± 0.1276	0.7691 ± 0.0652

**Table 2:** Quantitative descriptors derived from the CWT scalograms for the no-void and void conditions at FBG1 and FBG2.

Condition	Scalogram Entropy	Energy Concentration	High/Low-Band Energy Ratio
FBG1 No-void	12.381	0.70175	0.022325
FBG1 Void	12.401	0.69826	0.02672
FBG2 No-void	12.372	0.70033	0.02569
FBG2 Void	12.377	0.70112	0.026485

**Table 3:** Approximate frequency band mapping for the five-level DWT decomposition of the FBG strain response signal at a sampling rate of 2 kHz.

Component	Approximate Frequency Range (Hz)
D1	500–1000
D2	250–500
D3	125–250
D4	62.5–125
D5	31.25–62.5
A5	0–31.25

**Table 4:** Mean, standard deviation, and *p*-value of selected wavelet-energy features under the no-void and void conditions. Statistics were computed from 100 non-overlapping window-based observations per condition using a 0.05 s window length.

Feature	No-Void Mean	No-Void SD	Void Mean	Void SD	*p*-Value
FBG1 DWT-L2	0.42	0.0158	0.514	0.023	6.76 × 10^−5^
FBG1 DWT-L3	0.21	0.0158	0.28	0.0158	1.13 × 10^−4^
FBG2 WPT-N1	0.55	0.0158	0.67	0.0158	2.14 × 10^−6^
FBG2 WPT-N3	0.178	0.0084	0.26	0.0158	7.06 × 10^−6^

## Data Availability

The data that support the findings of this study are openly available at OST: https://doi.org/10.17605/OSF.IO/WVRB5.
